# Research capacity building integrated into PHIT projects: leveraging research and research funding to build national capacity

**DOI:** 10.1186/s12913-017-2657-6

**Published:** 2017-12-21

**Authors:** Bethany L. Hedt-Gauthier, Roma Chilengi, Elizabeth Jackson, Cathy Michel, Manuel Napua, Jackline Odhiambo, Ayaga Bawah, with input from the AHI PHIT Partnership Collaborative, Ahmed Hingora, Dominic Mboya, Amon Exavery, Kassimu Tani, Fatuma Manzi, Senga Pemba, James Phillips, Almamy Malick Kante, Kate Ramsey, Colin Baynes, John Koku Awoonor-Williams, Ayaga Bawah, Belinda Afriyie Nimako, Nicholas Kanlisi, Elizabeth F. Jackson, Mallory C. Sheff, Pearl Kyei, Patrick O. Asuming, Adriana Biney, Roma Chilengi, Helen Ayles, Moses Mwanza, Cindy Chirwa, Jeffrey Stringer, Mary Mulenga, Dennis Musatwe, Masoso Chisala, Michael Lemba, Wilbroad Mutale, Peter Drobac, Felix Cyamatare Rwabukwisi, Lisa R. Hirschhorn, Agnes Binagwaho, Neil Gupta, Fulgence Nkikabahizi, Anatole Manzi, Jeanine Condo, Didi Bertrand Farmer, Bethany Hedt-Gauthier, Kenneth Sherr, Fatima Cuembelo, Catherine Michel, Sarah Gimbel, Bradley Wagenaar, Catherine Henley, Marina Kariaganis, João Luis Manuel, Manuel Napua, Alusio Pio

**Affiliations:** 1 Partners In Health, Kigali, Rwanda; 2 Department of Global Health and Social Medicine, Harvard Medical School, Boston, MA 02115 USA; 3 Centre for Infectious Disease Research in Zambia, Lusaka, Zambia; 4 University of North Carolina at Chapel Hill, Chapel Hill, USA; 5 Heilbrunn Department of Population and Family Health, Mailman School of Public Health, Columbia University, New York City, USA; 6 Health Alliance International, Beira, Mozambique; 7 Beira Operational Research Center, National Institute of Health, Beira, Mozambique; 8 Regional Institute for Population Studies, University of Ghana, Accra, Ghana

**Keywords:** Research capacity strengthening, Research policy, Research funding, Africa, Health programs, Ghana, Mozambique, Rwanda, Tanzania, Zambia

## Abstract

**Background:**

Inadequate research capacity impedes the development of evidence-based health programming in sub-Saharan Africa. However, funding for research capacity building (RCB) is often insufficient and restricted, limiting institutions’ ability to address current RCB needs. The Doris Duke Charitable Foundation’s African Health Initiative (AHI) funded Population Health Implementation and Training (PHIT) partnership projects in five African countries (Ghana, Mozambique, Rwanda, Tanzania and Zambia) to implement health systems strengthening initiatives inclusive of RCB.

**Methods:**

Using Cooke’s framework for RCB, RCB activity leaders from each country reported on RCB priorities, activities, program metrics, ongoing challenges and solutions. These were synthesized by the authorship team, identifying common challenges and lessons learned.

**Results:**

For most countries, each of the RCB domains from Cooke’s framework was a high priority. In about half of the countries, domain specific activities happened prior to PHIT. During PHIT, specific RCB activities varied across countries. However, all five countries used AHI funding to improve research administrative support and infrastructure, implement research trainings and support mentorship activities and research dissemination. While outcomes data were not systematically collected, countries reported holding 54 research trainings, forming 56 mentor-mentee relationships, training 201 individuals and awarding 22 PhD and Masters-level scholarships. Over the 5 years, 116 manuscripts were developed. Of the 59 manuscripts published in peer-reviewed journals, 29 had national first authors and 18 had national senior authors. Trainees participated in 99 conferences and projects held 37 forums with policy makers to facilitate research translation into policy.

**Conclusion:**

All five PHIT projects strongly reported an increase in RCB activities and commended the Doris Duke Charitable Foundation for prioritizing RCB, funding RCB at adequate levels and time frames and for allowing flexibility in funding so that each project could implement activities according to their trainees’ needs. As a result, many common challenges for RCB, such as adequate resources and local and international institutional support, were not identified as major challenges for these projects. Overall recommendations are for funders to provide adequate and flexible funding for RCB activities and for institutions to offer a spectrum of RCB activities to enable continued growth, provide adequate mentorship for trainees and systematically monitor RCB activities.

**Electronic supplementary material:**

The online version of this article (10.1186/s12913-017-2657-6) contains supplementary material, which is available to authorized users.

## Background

Given limited human, financial and infrastructure resources, maximizing effectiveness of health programs in sub-Saharan Africa requires that policies and practice are based on evidence [1–6] and that interventions are monitored, evaluated and adjusted accordingly [3]. Despite this need for data-driven health programming, the corresponding knowledge production is limited and disproportionate in relation to the health burden the region bears [7–9]. Between 2003 and 2009, only 10% of health policy and systems research publications came from low- and middle-income countries (LMICs) [7]. A survey of research output of 847 health research institutions from 42 countries in sub-Saharan Africa in 2009 found an average of one peer reviewed publication per institution [10]. Inadequate research capacity in sub-Saharan Africa impedes the development and use of research to guide program implementation [3], heightening inequities in global health [11–14].

Despite the 1998 World Health Organization (WHO) call for the development of research policies and strategies to build national health research capacity in sub-Saharan African countries, research capacity building (RCB) efforts have faced numerous challenges [3, 13]. Most RCB activities in the region lack sufficient funding and thus are limited in depth and breadth [3, 11, 15]. Increasingly, agencies funding either research or program implementation with an evaluation component acknowledge the value of building national research capacity [16, 17]. However, funding from these agencies is often restricted, both in amount and timelines [18] and misaligned with national priorities [13], limiting an institution’s ability to develop RCB activities that address current needs.

In 2009, the Doris Duke Charitable Foundation’s (DDCF) African Health Initiative (AHI) began supporting the Population Health Implementation and Training (PHIT) Partnership projects [19]. The PHIT Partnership projects aimed to develop and implement interventions that linked implementation research and training to the delivery and evaluation of health care services. Projects in five countries – Ghana, Mozambique, Rwanda, Tanzania and Zambia – received funding for various activities, including RCB. In contrast to traditional restrictive funding mechanisms, PHIT projects included RCB funding in their budgets based on their program needs and goals. Throughout the five-year period covered in this paper, country projects had access to additional funding to promote more research capacity in the clinical and programmatic work. In this paper, leaders of RCB efforts in each of the five countries describe how their PHIT projects leveraged AHI funding for RCB activities, the achievements in RCB under this support, ongoing challenges, and lessons learned.

## Methods

### Description of the five PHIT projects

The five AHI-funded PHIT projects have been described in detail in other papers: Ghana [20], Mozambique [21], Rwanda [22], Tanzania [23] and Zambia [24]. In summary, PHIT projects were similar in their core principles but different in their specific activities, coverage and implementation partnerships [25, 26]. The primary goal for each project was strengthening the district-level health system [24–26]. However, focus on clinical and data quality improvement [25, 27], strengthening the health facility management [21, 22] and focus on the community-level of implementation [20, 22, 23] varied across countries. Further, the size of the implementation areas varied from Rwanda’s project covering two districts with 23 facilities to Mozambique’s project covering 13 districts with 146 facilities. Partnerships included Ministries of Health, local and international academic institutions and other non-governmental organizations.

RCB was a common denominator across the five country proejcts, strengthened by DDCF’s emphasis on implementation research and program evaluation [28]. However, each country differed on the amount of research already happening prior to the start of the PHIT project. A short description for each project’s research and RCB activities prior to AHI funding is provided below.

- The *Ghana* PHIT project was implemented in the Upper East region, which had a long history of health systems research. Prior to PHIT’s Ghana Essential Health Intervention Program (GEHIP), the Navrongo Health Research Centre had worked in the Upper East region for close to three decades, established a Navrongo Demographic Surveillance System and had successfully implemented a Community-based Health Planning and Services (CHPS) Program, which was adopted as a national blueprint for health delivery in Ghana. While other health system intervention programs had taken place in this region, PHIT’s GEHIP was the first comprehensive region-wide intervention that was anchored on the WHO health systems framework to assess the impact of the intervention on maternal and child health.
- The *Mozambique* PHIT project was implemented in the 13 districts of Sofala Province with support from Health Alliance International. Part of the implementation was to support the Beira Operations Research Center (CIOB). Created in 2005, CIOB is one of the three research centers of the Mozambique National Health Institute and is the only applied and implementation research center in the country. Prior to AHI funding, CIOB had limited staff and completed fewer studies. CIOB experienced substantial growth during the PHIT project.
- The *Rwanda* PHIT project was implemented in two districts where health care services had been managed by the Ministry of Health with support from a Boston-based non-governmental organization Partners In Health (PIH) since 2005. Prior to 2005, there was some research on health outcomes and service delivery in these two districts. However, the research was limited in scope, was primarily led by academic and clinical faculty from Harvard Medical School or Brigham and Women’s Hospital (institutional partners for PIH) and was without particular focus on national capacity building. The first coordinated efforts to establish research infrastructure and RCB activities began in 2010, catalyzed by AHI funding.
- In *Tanzania*, AHI funded a project called Connect that trained and deployed a new, paid cadre of community health workers, called Community Health Agents (CHAs), according to the Tanzanian Ministry of Health’s guidelines and policies. CHAs were deployed in three districts in two regions with existing health and demographic surveillance systems (HDSS) run by the Ifakara Health Institute (IHI). Prior to PHIT, IHI was already a successful research institution that utilized HDSS sites to make important contributions to public health through, for example, population-based research in malaria prevention and health systems strengthening. IHI’s HDSS in Morogoro and Pwani regions were deemed ideal platforms to conduct a randomized controlled trial on the impact of CHAs on child mortality. The Connect project began in 2011 with an AHI-funded posting of a demographer who provided continuous research mentoring to local junior scientists.
- In *Zambia*, the PHIT project was implemented by the Centre for Infectious Disease Research in Zambia (CIDRZ), a non-governmental research organization that was established in 2001. Before AHI funding, CIDRZ had established research in HIV and systems strengthening in support of the antiretroviral therapy (ART) program. The PHIT project used an integrated systems strengthening approach to improve the overall quality of 42 public facilities’ outpatient departments in three districts utilizing lessons learned from the ART work.

### Compiling information on PHIT-supported research capacity building activities

For this manuscript, each PHIT project nominated one to two member(s) of their team who led or coordinated the RCB activities completed using AHI funding. These individuals are all included as co-authors on this paper. For cohesion, we used Cooke’s framework to synthesize details about PHIT project activities specific to RCB [29]. This framework includes six dimensions of RCB: 1) building skills and confidence, 2) developing linkages and partnerships, 3) ensuring the research is “close to practice,” 4) developing appropriate dissemination, 5) investing in infrastructure, and 6) building elements of sustainability and continuity. The representatives responded to how much each of the RCB domains was a priority for AHI-funded RCB activities and provided a list of their specific activities that were linked to these domains via a semi-structured questionnaire (see Additional file 1). Co-authors Bethany Hedt-Gauthier, Jackline Odhiambo and Ayaga Bawah compiled responses, with follow-up skype interviews and emails to clarify questions arising from the completed survey. Responses from PHIT projects’ RCB leaders were collected and refined between September and December 2015.

In addition, respondents provided data on RCB activity inputs, outputs and outcomes indicators synthesized from other articles describing RCB monitoring and evaluation [29–31]. There were no standardized metrics for RCB activities across the five projects and projects only reported on RCB indicators when the data had been routinely collected during the implementation of the PHIT project. For this reason, projects at times had no data available for some specific indicators. When available, data could correspond to the overall PHIT project or to a specific RCB-activity implemented as part of the project. For each indicator, we report how many countries are represented and whether the countries are reporting for the combined project or single activity. The indicators were reported in aggregate for the 5 years of PHIT implementation as totals and averages.

Finally, each project key informant provided information on the degree to which common RCB challenges, as listed in a systematic review of non-academic RCB programs [30], persisted for their project and described any innovative activities made possible by AHI funding to alleviate these challenges. For each challenge, RCB leaders reported whether the challenge was a major challenge (score = 2), minor challenge (score = 1) or not a challenge (score = 0) and we reported the average response across the five PHIT projects.

### Ethics statement

This paper includes program descriptions from co-authors and data that is routinely collected through PHIT project monitoring and evaluation systems. As such, this paper falls under non-human subjects research.

## Results

### PHIT country projects’ approaches to research capacity building

**Fig. 1:**
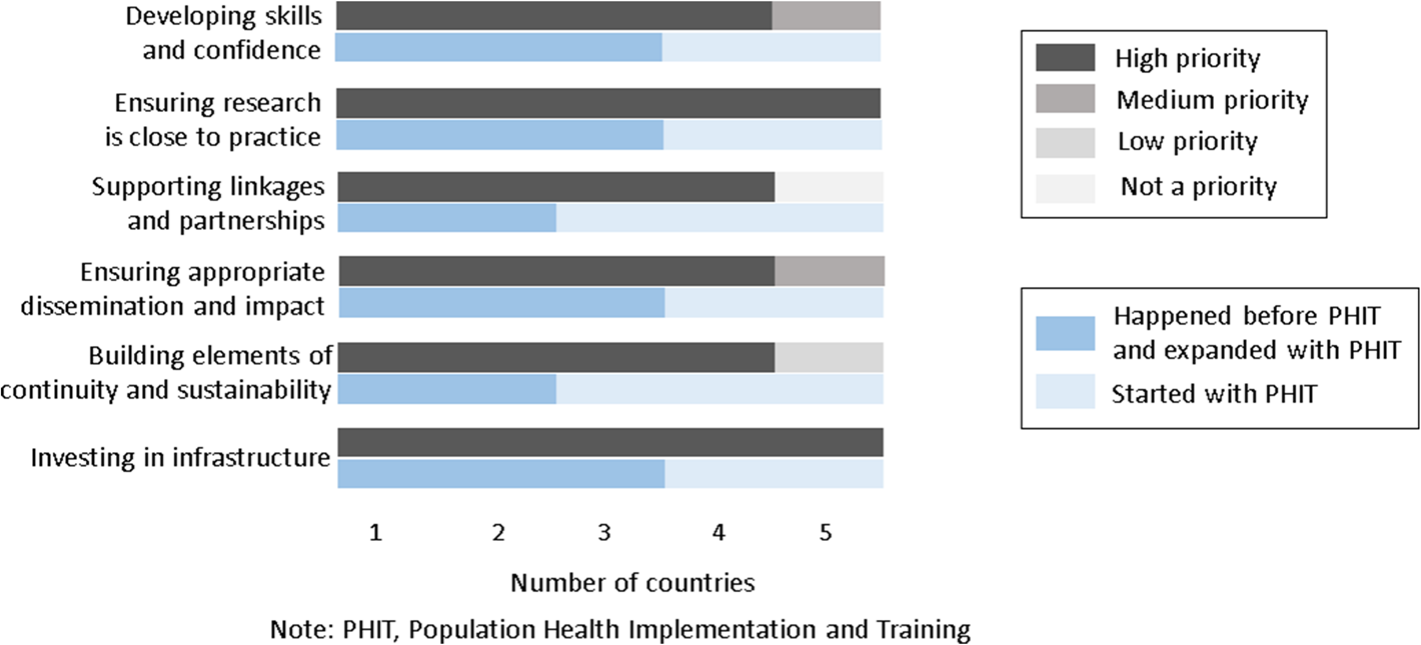
Research capacity building priorities and onset of activities for PHIT projects

In each of the Cooke’s domains, about half of the PHIT projects had some RCB activities happening prior to AHI funding and these activities were either expanded or catalyzed using the AHI funding (Fig. 1). Further, each program prioritized all five domains for RCB activities, and most country projects reported that each domain was a high priority (Fig. 1).

Specific RCB activities varied country-by-country. However, there were common themes to the types of activities supported using AHI funds (Table 1). Four country projects reported using AHI funds to provide scholarships for Masters and PhD degrees either at local or international institutions. All five projects used AHI funding to increase research administrative support and infrastructure. Four countries hired either research or administrative staff and four country projects used funding to construct or renovate spaces used for data collection, research and/or research training.

**Table 1:** Summary of key research capacity building activities for each PHIT country

Activities	Cooke’s domains impacted by this activity	Countries and Institutions				
		Ghana	Mozambique	Rwanda	Tanzania	Zambia
Scholarships	1. Skills and confidence 3. Linkages/Partnerships 5. Continuity and sustainability	- Masters and PhD degrees at international (UoB, NYU, AUoB, OSU) and local universities (UoG).	- Masters and PhD at local (UEM, UCM) and international (UW) universities	- Masters and PhD degrees at a local university (UoR)	- No direct scholarships but graduate research assistantship or salary support for masters and PhD degrees (CU and BU) and additional training for junior scientists at international universities (CU).	- Masters and PhD degrees at local (UoZ), regional (UoC) and international universities (LSHTM)
Administrative support and infrastructure	5. Continuity and sustainability 6. Investing in infrastructure	*Hiring staff* - Hired research staff (postdoctoral researcher and senior research scientists), administrative staff (accounting and clerical staff) and co-financed staff salaries *Building/renovating space* - Built office block for project staff *Procuring resources* - Purchased vehicles and motor cycles which went to DHMTs/RHMT - Purchased portable computers and GIS equipment for research	*Hiring staff* - Hired research staff (research assistants) to work side by side with the National Institute of Health and coordinate research activities; temporary data collectors and consultant to strengthen administrative structure of CIOB *Building/renovating space* - Supported partial running costs of health centers and small renovations *Procuring resources* - Bought computers for staff both program and admin. - Covered internet costs at research center	*Hiring staff* - Hired research staff (data collectors, coordinators, director of research) to provide administrative and logistic support to ongoing research projects *Building/renovating space* - Built a physical space for Research Department	*Hiring staff* - Hired research staff (for full time research roles including senior demographer to support data collectors with baseline and endline study implementation *Other* - Supported partial running costs of IHI research services such as demographic surveillance and data entry	*Building/renovating space* - Built rooms/ bought container spaces/ renovated rooms at >40 health facilities to create space for improved filing of medical records *Procuring resources* - Installed satellite dishes in facilities to improve internet access for data transfer - Installed data servers and bought computers for data entry
Mentorship	1. Skills and confidence 3. Linkages / Partnerships 4. Appropriate dissemination and impact 5. Continuity and sustainability	*Hiring/coordinating mentors* - Local mentors from the institution provided continuous mentorship *Coordinated mentorship activities* - Structured mentorship through several analysis and writing workshops	*Hiring/coordinating mentors* - Local mentors from the CIOB provided continuous mentorship - In-country technical advisors from HAI - Teaching assistant based out of UW *Supporting local universities* - Teaching select courses in the Masters of Public Health at local universities (UCM and UEM)	*Hiring/coordinating mentors* - Local mentors from PIH/IMB provided continuous mentorship - In-country technical advisor from HMS - Local mentors through UoR partnership - Long distance mentors through HMS partnership *Coordinated mentorship activities* - Structured mentorship through several 2-day writing workshops *Supporting local universities* - Teaching select courses at UoR	*Hiring/coordinating mentors* - Connect project PhD demographer resident at IHI provided continuous mentoring in quantitative research to junior scientists - Local mentor from IHI provided continuous mentorship in qualitative research - Long distance mentors through CU partnership *Coordinated mentorship activities* - Structured mentorship through Research Writing Workshop of several months duration	*Coordinated mentorship activities* - Structured mentorship through week-long writing workshops - Scheduled QI mentorship activities monthly at all the sites
Trainings	1. Skills and confidence 3. Linkages / Partnerships 5. Continuity and sustainability	*Short trainings* ^a^ - Series of trainings to develop and implement baseline survey - Monthly and quarterly data officers training - Knowledge management trainings through DDCF small grants *Long trainings* ^b^ - Cross-country PHIT led trainings on multilevel modelling and economics	*Short trainings* - Annual 2-week Operations Research training *Long trainings* - Annual data validation study led by junior researchers with on the job training - Training by doing on select data analysis techniques (qualitative and quantitative), research grant application and abstract writing workshops	*Short trainings* - Trainings on Complex Survey Analysis and Time Series Analysis with support to publication - Several 2-day scientific writing workshops *Long trainings* - Some related projects sent for an 8 months Operations Research training run by PIH/IMB	*Short trainings* - Weekly on the job trainings to develop and implement baseline survey - Trainings on qualitative and quantitative data analysis *Long trainings* - Research writing workshops for several months	*Short trainings* - Multi-level statistical modeling training through regional partnership by CUB - Semi-annual three-week training on QI - A 3-week training on leadership and management at UoA, USA *Long trainings* - Lean Six Sigma QI training and QI champions training through partnership with CGLUNC
Dissemination support	1. Skills and confidence 2. Research is close to practice 4. Appropriate dissemination and impact	*Dissemination trainings* - Training on dissemination to policy makers *Conference/Workshops* - Communications workshop *Publications* - Peer reviewed journals - Monthly publication on what works/what fails series	*Dissemination trainings* - Training on presentation for national and international audiences *Conference/Workshops* - Conference scholarships - Writing workshops for publication *Publications* - Peer reviewed journals	*Conference/Workshops* - Conference scholarships - Writing workshops for publication *Publications* - Peer reviewed journals	*Conference/Workshops* - Conference scholarships - Writing workshops for publication *Publications* - Peer reviewed journals	*Conference/Workshops* - Conference scholarships - Writing workshops for publication - Dissemination meetings at the Ministry of Health *Publications* - Peer reviewed journals

^a^Short trainings lasted for up to 1 month for each training sessions regardless of frequency of the training; ^b^Long trainings lasted for more than 1 month regardless of frequency of the training; *AUoB* American University of Beirut, *CIOB* Beira Operations Research Center, *BU* Brown University, *CGLUNC* Centre for Global Learning at University of North Carolina, *CU* Columbia University, *CUB* Catholic University in Beira, *UCM* Catholic University of Mozambique, *DDCF* Doris Duke Charitable Foundation, *DHMTs* District Health Management Teams, *UEM* Eduardo Mondlane University, *GIS* Geographic Information System, *HMS* Harvard Medical School, *LSHTM* London School of Hygiene and Tropical Medicine, *NYU* New York University, *PhD* Doctor of Philosophy, *PHIT* Population Health Implementation Training, *PIH/IMB* Partners In Health/Inshuti Mu Buzima, *QI* Quality Improvement, *RHMTs* Regional Health Management Teams, *UoA* University of Alabama, *UoB* University of Basel, *UoC* University of Capetown, *UoR* University of Rwanda, *UW* University of Washington, *UoZ* University of Zambia, *UoG* University of Ghana, *OSU* Ohio State University, *USA* United States of America

All five country projects reported using funding to support mentorship activities (Table 1). Four PHIT projects used funding to hire mentors or facilitate mentorship from national researchers, from international researchers locally based or from international researchers using a distance-mentorship model. For the distance-mentorship model, approaches ranged from exclusively relying on email exchanges with periodic calls to a blended model that included these distant exchanges complemented with occasional face-to-face meetings, most often when the international researcher was visiting the project to support other aspects of the work. Four PHIT projects offered writing workshops to consolidate mentorship activities and accelerate dissemination. Finally, two PHIT projects used AHI funding to provide support to local universities by teaching courses within their academic programs.

All PHIT projects used AHI funding to implement research trainings to build skills. The target competencies, length and style of training varied by program (Table 1). Often PHIT projects implemented multiple training activities of increasing complexity to allow an emerging researcher’s skills to grow over the training series. Finally, all five country projects used AHI funding to support research dissemination, including scholarships for conferences (four projects) and writing workshops with the goal of producing scientific publications (four projects).

### Outputs, outcomes and impacts of PHIT research capacity building activities

Table 2 synthesizes indicators across the five PHIT projects, indicating how many projects had data available and the scope of that data. A country project-level summary is available in an online supplementary table (see Additional file 2). Over the 5 years, there were 201 participants in RCB-related trainings (Table 2). Though rarely documented, 14 of these were known to be practitioners or clinical staff and 27 were programmatic staff. For the four PHIT projects with data available, 22 PhD, Masters or research scholarships were awarded using AHI funds. Three country projects had recorded data relevant to research mentorships, documenting 56 research mentor-mentee pairings. Forty-four research protocols were documented, and 116 manuscripts were attributed to the PHIT projects. Over the 5 years, for the four country projects that tracked this data, there were 59 peer-reviewed publications, of which 29 (49.2%) were first authored by a national researcher and 18 (30.5%) had a national senior author. Four PHIT projects reported that trainees presented at 99 conferences, workshops, or public lectures. Although very few PHIT projects systematically recorded this information, 43 trainees were documented to collaborate on new research projects, 4 trainees were documented to be leading new research projects and 19 trainees went on to become facilitators for a training course or mentors.

**Table 2:** Metrics of outputs, outcomes and impacts of PHIT research capacity building activities

		Countries Reporting			
Capacity building level	Indicators	Overall	For specific activities	Not collecting data	Aggregate Outcomes ^a^
Individuals / Teams	Average number of applications per training per country	2	2	1	18
	Average number of participants per training per country	2	2	1	12
	Number of participants completing the training	2	2	1	201
	Number of practitioners/clinical staff trained	2	0	3	14
	Number of program staff trained	2	0	3	27
	Number of PhD, Masters or research scholarships awarded	2	2	1	22
	Number of mentorship relationships (individual or team based)	2	1	2	56
	Number of research protocols developed	3	1	1	44
	Number of publishable manuscripts written	3	1	1	116
	Number of peer reviewed publications	3	1	1	59
	Number of peer reviewed publications with a national first author	3	1	1	29
	Number of peer reviewed publications with a national senior author	3	1	1	18
	Number of conferences / workshops / public lectures where trainees presented	2	2	1	99
	Number of trainees collaborating in new research	2	1	2	43
	Number of trainees who led new research projects	2	0	3	4
	Number of trainees who became facilitators or mentors	0	3	2	19
Organizational	Number of research trainings conducted (whether long or short)	3	2	0	54
	Range of the main trainings in days per session	5	0	0	2–14 days
	Range of the training contact time in days	2	3	0	2–150 days
	Total number of PhD / Masters level trainers	4	1	0	25
	Number of national PhD or Masters trainers / facilitators	4	0	1	11
	Number of research related career promotions	2	0	3	9
	Number of research guidelines used (internal, government or network	1	1	3	8
Networks	Number of networks/ collaborations established or joined	1	1	3	9
	Number of forums between policy makers and researchers	2	2	1	37
	Number of times research findings impacted program, practice or policy	0	1	4	3
	Number of times research findings impacted quality of health care or health outcomes	0	0	5	N/A
	Number of times research findings led to reduction in costs of product, service or intervention	0	0	5	N/A
	Number of external donors expressing interest to fund activities	1	2	2	8

^a^Calculated as total for countries that reported the indicator unless the indicator specifies “average” and then is an average across countries. *QI* Quality improvement

At the organizational level, 54 research trainings were held using AHI funding, ranging from 2 to 14 in-person training days with 2–150 days of additional contact (practicums with additional mentorship). Twenty-five PhD- or Masters-level trainers were available for RCB trainings, of whom at least 11 were national trainers. Research promotions were rarely tracked, and only nine promotions were documented and attributed to the PHIT-related trainings. Only two PHIT projects reported eight different guidelines developed or used as part of the AHI funding.

In terms of using the AHI funding to build research networks, for the two PHIT projects with data available, 9 new networks and collaborations were established. Four PHIT projects reported holding 37 forums between policy makers and researchers. Only one country had routinely collected data on the number of research projects that impacted policy and no PHIT projects had documentation on the frequency that research findings impacted quality of care or outcomes or reduced costs of programs/service. For the three PHIT projects reporting, eight additional external donors had expressed interest to fund research and/or RCB activities.

### Ongoing challenges and novel solutions for research capacity building

Common challenges for RCB activities were grouped into five categories – trainee background; mentorship, teaching and trainee support; infrastructure and logistics; institutional support and buy-in; and sustainability and funding (Table 3). For trainee background, the most noted challenge was ability to publish in international journals, which respondents linked to the fact that trainees were best suited to produce research close to practice, which is often difficult to publish. In addition, for several PHIT projects, English was not the first language for trainees, hence a language barrier in research writing, and few had any previous experience in scientific writing. The PHIT projects addressed these challenges by identifying journals that published operational and implementation research, providing language and writing support to trainees, and “co-producing” research, with writing teams including implementers and researchers. While PHIT projects reported having mismatches between trainees’ skills and initial training goals, they also noted the importance of tailoring training goals to the abilities of the trainees, offering a range of activities to enable different levels of skills building and using advanced trainees to provide support to less experienced trainees.

**Table 3:** Challenges faced by country RCB programs and recommended solutions

Challenges	Level of challenge^a^	Solutions
Trainee backgrounds		
Language barriers	1.0	*Develop/strengthen internal resources:* - Strong English speakers on the team to review/edit materials - Work with trainees/ teams to improve English skills - Support groups / workshops e.g. writing workshop, analysis clinic *Adjust according to trainee background:* - Re-orient training priorities to match trainee baseline skills - Have a spectrum of activities for different skills levels so trainees can start at appropriate levels and grow in the program - Use those with advanced in skills as Research / Training Assistants or trainers *Increase publication success:* - Seek journals that welcome operational research - Increase writing mentorship to get to publication quality - Pair trainees with mentors for intensive support - Publish in local language then translate to English
Mismatch between participants capabilities and training priorities	1.2	
Difficulty managing groups of different academic levels	0.8	
Difficulty for participants to publish in international journals	1.6	
Mentorship, teaching and trainee support		
Inability of mentors to follow-up due to high need of mentorship	1.4	*Prioritize mentorship:* - Recruit and fund mentors primarily for RCB activities - Build mentorship into RCB activities - Leverage long distance mentors through phone / skype meetings and email reviews but ideally still have periodic face to face mentorship *Make mentorship efficient:* - Encourage group mentorship e.g. writing retreats - Project managers/coordinators to track mentorship needs to reduce stalling of projects that need further help
Participant drop out due to lack of mentorship	1.0	
Poor communication between participants and supervisors	0.6	
Infrastructure and logistics challenges		
Difficulty in securing adequate space for RCB activities	0.0	*Prioritize funding infrastructure / logistics* - Rent training space/ build training center - Pay for participants transport - Budget for necessary materials e.g. portable internet modems, satellite internet installation, data analysis software
Difficulty for participants in accessing training location	0.0	
Inadequate materials for participants to complete research	0.8	
Poor internet for participants	1.2	
Poor internet for facilitators/mentors	0.4	
Institutional support and buy-in		
Difficulty getting buy-in for RCB activities from institutions	0.6	*Make RCB integrated with a budget line and dedicated staff* *Cultivate RCB as organizational core ethos* - Develop research guidelines that outline capacity building as a component for all research activities - Train leaders to be consumers of research *Seek support from the Ministry of Health for increased collaboration and support of employee participation*
RCB initiatives blocked by leadership	0.0	
Competing work responsibilities for participants	1.4	
Sustainability and funding challenges		
Participant drop out due to changing employment	1.6	*Employ trainee retention strategies* - Invest in professional development such as scholarships - Allow continued involvement despite attrition as long as trainee stays meaningfully engaged *Use trainee involvement strategies* - Pay for percent time for research - Align research to be seen as part of work *Broaden network of support / funding* - Seek and advocate for government support/funding - Develop training programs within established institutions such as universities - Seek for specific funding pools that support RCB - Plan for long-term training programs
Dependence on external institutions or donors for funding	1.6	
Donors don’t want to fund these types of activities	0.8	

^a^Reported as an average of the country responses. Each country reported 0 = Not a challenge; 1 = Minor challenge and 2 = Major challenge. Higher score indicates the issue was identified as a bigger challenge or by more countries as a challenge

All PHIT projects reported that the demand for mentorship exceeded the mentorship resources available (Table 3). The pool of available mentors for each PHIT project was small. Leveraging long-distance mentors expanded the number of mentors available, but introduced additional challenges of providing adequate support to new researchers when language and communication infrastructure barriers existed. Trainee drop-out due to inadequate mentorship was an ongoing challenge. Respondents noted that provision of mentorship was a high priority for AHI-funding and most PHIT projects used formal group skills-building activities, such as writing workshops, to efficiently spread the mentorship resources.

Many of the infrastructure and logistics challenges reported in RCB activities in other settings were not challenges to the AHI-funded projects. All respondents attributed the absence of these barriers to the ability to flexibly target funds from AHI to strengthen or build research infrastructure and procure necessary research materials where gaps were anticipated or emerged during the grant period. The most common challenge in this domain was poor internet. While many of the PHIT projects found work-arounds such as cell-phone modems, all PHIT projects noted that internet availability limited the effectiveness of RCB activities. Similarly, many of the institutional buy-in challenges for RCB activities seen in other settings were not reported by PHIT projects. Respondents attributed the ease of buy-in to the fact that PHIT projects were collaborative across all institutions, including local research institutions, and that research was closely linked to the trainee’s work and identified priorities. However, competing work demands was a noted challenge for trainees and an area where no novel solutions were provided by respondents.

PHIT projects reported sustainability and funding of RCB activities as the largest ongoing challenge (Table 3). Trainees dropped out, during or after the training, due to changing employment especially when the AHI funding was coming to an end and new funding for research or RCB had not been secured. PHIT projects did not identify donors not wanting to fund RCB activities as a large issue; however, dependence on external funding for RCB activities was a major challenge. PHIT projects are currently trying to expand their funding sources for research and RCB activities to continue after the end of the AHI grants and to continue to link RCB activities to existing institutions in-country and at the US partners’ home institutions. All respondents commended DDCF for the level of funding and the flexibility of funding for RCB activities and recommended that other granting organizations follow suit.

## Discussion

In synthesizing the RCB experiences across the five PHIT country projects, messages for two key stakeholders emerged. The first is for the programs implementing RCB activities on how to strategically leverage funds to address common challenges through innovative solutions. The second message is to the funders of RCB activities on how their funding can best support a culture of effective and country-focused RCB. In the process, six key lessons emerged across all PHIT projects.

### Key lesson 1: For RCB to occur, funders need to provide support and the support needs to be flexible to reflect context and local capacity.

Prior to DDCF funding, all five country projects had difficulties obtaining sufficient resources for specific RCB activities. However, the availability and flexibility of AHI resources enabled each PHIT project to implement the suite of activities that matched its needs. Funding with small budgets or restricted timelines encourage one-off training programs without the needed support, particularly mentorship, to build national research capacity [18]. Funding agencies should make sufficient and flexible resources available for each program to develop the most appropriate plan for their current research landscape. Further, funding timelines should span years to reflect that RCB requires a long term commitment to be most effective.

### Key lesson 2: Effective research capacity building programs should include a continuum of activities.

Funding restrictions and limited number of trainers also encourage isolated RCB training programs that target only one skill [30, 32, 33]. This model fails to facilitate the translation of research skills into research projects after the training [30, 34]. Flexible DDCF funding through AHI allowed the PHIT projects to implement a spectrum of RCB activities, in parallel and sequentially, which reflected local context and range of baseline capacities. Individuals started with trainings appropriate for their skill levels and continued to develop skills until research independence. This also created an environment where individuals more advanced in the training spectrum could support and provide mentorship to the more junior trainees, increasing the quantity of training and mentorship possible with limited resources.

### Key lesson 3: Use research and research capacity building funds to strengthen existing research institutions when possible.

For four PHIT projects, existing national research institutions were at the core of their projects’ research. These projects noted that building on existing infrastructure accelerated the initiation of projects and enabled the continuation of research implementation and development of research skills outside of the scope of PHIT-specific projects. Building on existing institutions has been a noted RCB challenge [35]; however, it promotes the sustainability of research capacity [8, 36] and can ensure the continuation of activities even after the initial funding has ended.

### Key lesson 4: Research that is a focus of capacity building activities should be closely linked to health program implementation.

Linking PHIT-related research to implementation, program evaluation and quality improvement enhanced the commitment of trainees to the training process until the completion of the training deliverables (most often, a research paper). The connection between research and challenges and priorities encountered during work also increases the likelihood of the research influencing policy and practice [1, 29, 37, 38]. For the PHIT projects, it also reduced the perception that the research training was in competition with the individual’s work, making the trainees and their host institutions more willing participants. While trainees faced publication and time constraint challenges, solutions such as identifying implementation research focused journals and having protected research and writing time have been proposed [29].

### Key lesson 5: Mentorship is critical, but provision of mentorship may need to be creative.

Mentorship is critical for developing capacity [39, 40]; however, it is often lacking in RCB activities in LMICs [38, 41, 42]. While each PHIT project noted the essential role of mentorship for their RCB activities, mentorship resources were limited requiring creativity in supporting trainees. All PHIT projects used some form of “learning-by-doing”, including “deliverable-driven” training models that provided trainees opportunities to learn and immediately apply concepts while receiving mentorship, resulting in tangible products such as a protocol or paper. This hands-on mentorship fosters confidence with the key research concepts and skills [39, 40]. PHIT projects also created mentorship groups, pairing teams of trainees with one dedicated mentor to complete a deliverable, expanding mentorship resources and facilitating peer-to-peer learning. Finally, many PHIT projects used e-mentoring, linking in-country trainees with international researchers within their network.

### Key lesson 6: Measure and monitor research capacity building outputs and impacts.

Ongoing measuring and monitoring of RCB activities is vital to ensure effective implementation of RCB activities [29, 34, 36]. All PHIT projects monitored some aspects of their RCB work and many noted that the ability to report on these helped advocate for additional resources. However, a weakness of the RCB implementation was that metrics were not standardized and what was collected varied from site-to-site. To date, no internationally agreed upon metrics exist and we recommend that a standardized tool of RCB indicators be developed and required by funders for future grants with an emphasis on RCB. This standardized tool should include clear indicator definitions and modes for data collection, particularly for more abstract concepts, such as “number of research projects that impacted policy.” PHIT projects also suggested that more of these indicators of RCB activities assess long-term impact and sustainability.

## Conclusion

All five PHIT projects strongly reported an increase in RCB activities and commended DDCF for prioritizing RCB, funding RCB at adequate levels and time frames and for allowing flexibility in funding so that each project could implement activities according to their trainees’ needs. As a result, many common challenges for RCB, such as adequate resources and local and international institutional support, were not identified as major challenges for these projects. The overall design of the PHIT projects, which mandated close partnership with local institutions, also set a culture which included RCB at the local institutional as well as individual level. These partnerships also ensure the sustainability of the research and research capacity building initiatives as the individuals with increased skills will continue to exist and grow within these local institutions.

However, some common challenges persisted, most notably the challenge of adequate mentorship capacity to meet demands. As RCB programs mature, more national mentors will be available to expand this work. In the meantime, other programs with some funding for RCB can look at the successes and challenges of these five PHIT projects for inspiration on how to maximize RCB using creative mentorship models and how to provide a myriad of training activities to ensure continuous skills growth on teams.

## Additional files

Additional file 1: PHIT Cross-site paper on Research Capacity Building Data Collection Form Round 1 of Data Collection. (DOCX 36 kb)

Additional file 2: Metrics table outlining each country’s indicator measurement. (DOCX 32 kb)

## Abbreviations

AHI: African Health Initiative
ART: Anti-retroviral therapy
CHAs: Community Health Agents
CIDRZ: Center for Infectious Disease Research Zambia
CIOB: Beira Operations Research Center
DDCF: Doris Duke Charitable Foundation
GEHIP: Ghana Essential Health Intervention Program
HDSS: Health and demographic surveillance system
HIV: Human immunodeficiency virus
IHI: Ifakara Health Institute
LMICs: Low and middle income countries
PHIT: Population Health Implementation and Training partnerships
PIH: Partners In Health
RCB: Research capacity building
WHO: World Health Organization

## References

[1] Yamey G, Feachem R (2011). Evidence-based policymaking in global health – the payoffs and pitfalls. Evidence-Based Med.

[2] Birbeck GL, Wiysonge CS, Mills EJ (2013). Global health: the importance of evidence-based medicine. BMC Med.

[3] Kirigia JM, Wambebe C (2006). Status of national health research systems in ten countries of the WHO African region. BMC Health Serv Res.

[4] Pang T, Sadana R, Hanney S (2003). Knowledge for better health — a conceptual framework and foundation for health research systems. Bull World Health Organ.

[5] Bissell K, Lee K, Freeman R (2011). Analysing policy transfer: perspectives for operational research. Int J Tuberc Lung Dis.

[6] Uneke CJ, Edeoha AE, Ndukwe CD (2013). Research priority setting for health policy and health systems strengthening in Nigeria: the policy makers and stakeholders’ perspective and involvement. PanAfrican Med J.

[7] Adam T, Ahmad S, Bigdeli M, Ghaffar A, Røttingen JA (2011). Trends in health policy and systems research over the past decade: still too little capacity in low-income countries. PLoS One.

[8] Kellerman R, Klipstein-Grobusch K, Weiner R, Wayling S, Fonn S (2012). Investing in African research training institutions creates sustainable capacity for Africa: the case of the University of the Witwatersrand School of public health masters programme in epidemiology and biostatistics. Health Res Policy Syst.

[9] Langer A, Díaz-olavarrieta C, Berdichevsky K, Villar J (2004). Why is research from developing countries underrepresented in international health literature, and what can be done about it?. Bull World Health Organ.

[10] Kebede D, Zielinski C, Mbondji PE (2014). Research output of health research institutions and its use in 42 sub-Saharan African countries: results of a questionnaire-based survey. J R Soc Med.

[11] Lansang MA, Dennis R (2004). Building capacity in health research in the developing world. Bull World Health Organ.

[12] Airhihenbuwa CO, Shisana O, Zungu N (2011). Research capacity building: a US-south African partnership. Glob Health Promot.

[13] Nuyens Y. 2005. No Development Without Research: A challenge for capacity strengthening. Global Forum for Health Research. [Online]. http://announcementsfiles.cohred.org/gfhr_pub/assoc/s14828e/s14828e.pdf. Accessed 4 Mar 2016.

[14] Tugwell P, Sitthi-Amorn C, Hatcher-Roberts J (2006). Health Research profile to assess the capacity of low and middle income countries for equity-oriented research. BMC Public Health.

[15] Masukume G (2012). A 54 year analysis of articles from Mpilo central hospital, Bulawayo, Zimbabwe - 168 articles cited 999 times. Malawi Med J.

[16] National Institute of Health. 2010. RePORT: Building Global Health Research Capacity. [Online]. https://www.niehs.nih.gov/research/assets/docs/building_global_health_research_capacity_508.pdf. Accessed 8 Dec 2015.

[17] Medical Research Council. 2015. Funding: Health systems initiative research call 3. [Online]. https://www.mrc.ac.uk/funding/browse/hsri-call-3/health-systems-research-initiative-call-3/. Accessed 8 Dec 2015.

[18] Bennett S, Corluka A, Doherty J, Tangcharoensathien V (2012). Approaches to developing the capacity of health policy analysis institutes: a comparative case study. Health Res Policy Syst.

[19] African Health Initiative. Population Health Implementation and Training Partnerships. [Online]. http://www.ddcf.org/what-we-fund/african-health-initiative/. Accessed 22 Dec 2015.

[20] Awoonor-Williams JK, Bawah AA, Nyonator FK (2013). The Ghana essential health interventions program: a plausibility trial of the impact of health systems strengthening on maternal & child survival. BMC Health Serv Res.

[21] Sherr K, Cuembelo F, Michel C (2013). Strengthening integrated primary health care in Sofala Mozambique. BMC Health Serv Res.

[22] Drobac PC, Basinga P, Condo J (2013). Comprehensive and integrated district health systems strengthening: the Rwanda population health implementation and training (PHIT) partnership. BMC Health Serv Res.

[23] Ramsey K, Hingora A, Kante M (2013). The Tanzania connect project: a cluster randomized trial of the child survival impact of adding paid community health workers to an existing facility-focused health system. BMC Health Serv Res.

[24] Stringer JSA, Chisemble-Taylor A, Chibwesha CJ (2013). Protocol-driven primary care and community linkages to improve population health in rural Zambia: the better health outcomes through mentoring and assessment (BHOMA) project. BMC Health Serv Res.

[25] Hirschhorn LR, Baynes C, Sherr K (2013). Approaches to ensuring and improving quality in the context of health system strengthening: a cross-site analysis of the five African health initiative partnership programs. BMC Health Serv Res.

[26] Bryce J, Requejo JH, Moulton L (2013). A common evaluation framework for the African health initiative. BMC Health Serv Res.

[27] Mutale W, Chintu N, Amoroso C (2013). Improving health information systems for decision making across five sub-Saharan African countries: implementation strategies from the African health initiative. BMC Heal Serv.

[28] Bassett MT, Gallin EK, Adedokun L, Toner C (2013). From the ground up: strengthening health systems at district level. BMC Health Serv Res.

[29] Cooke J (2005). A framework to evaluate research capacity building in health care. BMC Fam Pract.

[30] Mugabo L, Rouleau D, Odhiambo J (2015). Approaches and impact of non-academic research capacity strengthening training models in sub-Saharan Africa: a systematic review. Health Res Policy Syst.

[31] Banzi R, Moja L, Pistotti V, Facchini A, Liberati A (2011). Conceptual frameworks and empirical approaches used to assess the impact of health research: an overview of reviews. Health Res Policy Syst.

[32] Ajuwon AJ, Kass N (2008). Outcome of a research ethics training workshop among clinicians and scientists in a Nigerian university. BMC Med Ethics.

[33] Mbuagbaw L, Wiysonge CS, Nsagha DS, Ongolo-Zogo P, Pantoja T (2011). An introduction to systematic reviews and meta-analysis: a workshop report on promoting evidence based medical practice through capacity building in research synthesis. Pan Afr Med J.

[34] Bates I, Akoto AYO, Ansong D (2006). Evaluating health research capacity building: an evidence-based tool. PLoS Med.

[35] Bates I, Taegtmeyer M, Squire SB (2011). Indicators of sustainable capacity building for health research: analysis of four African case studies. Health Res Policy Syst.

[36] Minja H, Nsanzabana C, Maure C (2011). Impact of health research capacity strengthening in low- and middle-income countries: the case of WHO/TDR programmes. PLoS Negl Trop Dis.

[37] Zachariah R, Harries AD, Ishikawa N (2009). Operational research in low-income countries: what, why, and how?. Lancet Infect Dis.

[38] Zachariah R, Reid T, Srinath S (2011). Building leadership capacity and future leaders in operational research in low-income countries : why and how?. Int J of Tuberc Lung Dis.

[39] Ramsay A, Harries AD, Zachariah R (2014). The structured operational research and training initiative for public health programmes. Public Health Action.

[40] Harries AD, Marais B, Kool B (2014). Mentorship for operational research capacity building: hands-on or hands-off?. Public Health Action.

[41] Zachariah R, Reid T, Ford N (2012). The 2012 world health report “no health without research”: the endpoint needs to go beyond publication outputs. Tropical Med Int Health.

[42] Ghaffar A, Ijsselmuiden C, Zicker F. 2010. Changing Mindsets: Research capacity strengthening in low- and middle-income countries. Council on Health Research for Development, Global Forum for Health Research, Special Programme for Research & Training in Tropical Diseases. [Online]. http://www.who.int/tdr/publications/tdr-research-publications/changing_mindsets/en/. Accessed 19 Oct 2015.

