# Supplementary material for: Research capacity building integrated into PHIT projects: leveraging research and research funding to build national capacity
Source: BMC Health Serv Res. 2017 Dec 21;17(Suppl 3):825. doi: 10.1186/s12913-017-2657-6 (PMC5763288; doi:10.1186/s12913-017-2657-6)
Supplement: Supplementary file 1 — PHIT Cross-site paper on Research Capacity Building Data Collection Form Round 1 of Data Collection. (DOCX 36 kb) [file 12913_2017_2657_MOESM1_ESM.docx]

**Additional File 1:**

**PHIT Cross-site paper on Research Capacity Building**

**Data Collection Form**

**Round 1 of Data Collection**

**Country Name: ____________________________**

**Background:** The purpose of this form is to collect data on the research capacity building activities supported under PHIT/DDCF. These activities can often be difficult to tease out, since in country, there are multiple partners engaging in PHIT activities and some activities are “PHIT-related” and others are not. As much as possible, we want information on PHIT-related activities – so activities funded by PHIT or reported out as PHIT-activities. Also, “activities” is broadly defined as an event or action that was intended directly or indirectly to build research capacity.

**Informants:** For initial data collection, the co-authors will respond on behalf of their country teams. However, a summary of the co-author responses will be provided and they can engage other team members to refine/update/complete responses. For the purpose of facilitating review, highlight your response in yellow.

**Overview:** There are 5 major sections to this initial data collection:

1. Section A: Summary of up to five major research capacity building activities.
2. Section B: Overview of sub-activities undertaken, using Cooke’s framework categories.
3. Section C: Challenges/barriers to RCB
4. Section D: M&E of RCB activities
5. Section E: Plans moving forward
6. Describe up to five of the major research capacity building activities undertaken with PHIT funding. Note that these can be formal activities or provision of certain human resources, etc. If no activities have been undertaken, then leave the section below blank.

|  |  |
| --- | --- |
|  |  |
|  |  |
|  |  |
|  |  |

**Section B:** Co-authors of the PHIT Cross-site paper on research capacity building agreed to use the Cooke’s framework to describe and evaluate research capacity building. Cooke’s framework goes beyond outcome-based metrics to measure the process of research capacity building. The framework is based on the following six principles. For each principle, describe whether or not this priority and whether this was happening prior to PHIT funding, initiated during PHIT funding or not yet initiated.

For each section, respondent indicated whether:

- This was a priority (response options: high priority, medium priority, low priority or not a priority)
- This was newly initiated with PHIT (response options: Happening prior to PHIT and not expanded; Happening prior to PHIT and expanded with PHIT; Started with PHIT; Was not happening prior or during PHIT).

1. **Developing skills and confidence**: The development of research core competencies (protocol development, data analysis, manuscript writing etc) and sharing of research skills with others.

**Was this a priority:**

**Was this newly initiated with PHIT:**

**Comments:**

1. **Ensuring research is close to practice:** The focus on generation of knowledge that is relevant for service user, practice or policy concerns.

**Was this a priority:**

**Was this newly initiated with PHIT:**

**Comments:**

1. **Supporting linkages and partnerships**: The emphasis on networking, building partnerships, and collaborations for developing, exchanging and enhancing research skills and practice knowledge. This can be between academia and service organizations, different countries, different professional groups, researchers and policy makers etc.

**Was this a priority:**

**Was this newly initiated with PHIT:**

**Comments:**

1. **Ensuring appropriate dissemination and impact**: Making known research activities and outcomes to and beyond peer-review journals, including media, internet, publicity through factsheets etc.

**Was this a priority:**

**Was this newly initiated with PHIT:**

**Comments:**

1. **Building elements of continuity and sustainability**: Maintenance of the newly acquired skills and structures to undertake research and providing further opportunities for engagement.

**Was this a priority:**

**Was this newly initiated with PHIT:**

**Comments:**

1. **Investing in infrastructure**: Structures and processes set up to enable smooth and effective running of research projects.

**Was this a priority:**

**Was this newly initiated with PHIT:**

**Comments:**

**Section C:** What challenges or barriers did you face in research capacity building work under PHIT projects and how did you address them? (Categories: Not applicable, Not a challenge, Minor challenge, Major challenge)

|  | **Response** | **Comments?** |
| --- | --- | --- |
|  |  | **(If a challenge, did you have any solutions to these challenges? If Yes, please describe.)** |
| **RCB initiatives blocked by leadership** |  |  |
| **Difficulty getting buy-in for RCB activities from institutions** |  |  |
| **Participant drop out due to lack of mentorship** |  |  |
| **Participant drop out due to changing employment** |  |  |
| **Competing work responsibilities for participants** |  |  |
| **Poor communication between participants and supervisors** |  |  |
| **Poor internet for participants** |  |  |
| **Poor internet for facilitators/mentors** |  |  |
| **Inadequate materials for participants to complete research** |  |  |
| **Difficulty in securing adequate space for research capacity building activities** |  |  |
| **Difficulty for participants in accessing training location** |  |  |
| **Difficulty for participants to publish in international journals** |  |  |
| **Language barriers** |  |  |
| **Mismatch between participants capabilities and training priorities** |  |  |
| **Difficulty managing groups of different academic levels** |  |  |
| **Inability of mentors to follow-up due to high need of mentorship** |  |  |
| **Donors don’t want to fund these types of activities** |  |  |
| **Dependence on external institutions or donors for funding** |  |  |

**Section D:** With PHIT funding, did you monitor research capacity building activities?

☐ No ☐ Yes (If Yes, please continue below until section is completed)

For research capacity building activities under PHIT projects, what metrics did you use and what were the outputs/outcomes/impacts?

| **Metric** | **Not measured** | **Measured for overall program** | **Measured for specific activities** | **What was the output /outcome / impact for this measure?** |
| --- | --- | --- | --- | --- |
| Total number of research trainings conducted | ☐ | ☐ | ☐ |  |
| Number of different types of research trainings conducted | ☐ | ☐ | ☐ |  |
| Total number of PhD or Masters level trainers / facilitators | ☐ | ☐ | ☐ |  |
| Number of local PhD or Masters trainers / facilitators | ☐ | ☐ | ☐ |  |
| Average number of participants expressing research interest through applications for trainings | ☐ | ☐ | ☐ |  |
| Average number of participants accepted in research trainings | ☐ | ☐ | ☐ |  |
| Total number of people trained | ☐ | ☐ | ☐ |  |
| Number of practitioners / clinical staff trained | ☐ | ☐ | ☐ |  |
| Number of Program staff trained | ☐ | ☐ | ☐ |  |
| Duration of the main training in weeks | ☐ | ☐ | ☐ |  |
| Amount of training/contact time in days | ☐ | ☐ | ☐ |  |
| Amount of training/contact time in hours | ☐ | ☐ | ☐ |  |
| Number of mentorship relationships (individual-mentor or team-mentor) | ☐ | ☐ | ☐ |  |
| Average number of mentorship hours per mentorship relationship | ☐ | ☐ | ☐ |  |
| Number of research protocols written | ☐ | ☐ | ☐ |  |
| Total number of publishable manuscripts written | ☐ | ☐ | ☐ |  |
| Number of peer reviewed publications | ☐ | ☐ | ☐ |  |
| Number of local first authors | ☐ | ☐ | ☐ |  |
| Number of local last/senior authors | ☐ | ☐ | ☐ |  |
| Average impact factor for journals papers are submitted to | ☐ | ☐ | ☐ |  |
| Average download numbers of papers | ☐ | ☐ | ☐ |  |
| Average number of citations | ☐ | ☐ | ☐ |  |
| Number of conferences / workshop/ consultancies / public lectures | ☐ | ☐ | ☐ |  |
| Number of career promotions / research related jobs | ☐ | ☐ | ☐ |  |
| Number of PhD / Masters / research scholarships awarded | ☐ | ☐ | ☐ |  |
| Number of participants collaborating in new research projects | ☐ | ☐ | ☐ |  |
| Number of participants who became facilitators or mentors | ☐ | ☐ | ☐ |  |
| Number of participants leading new research projects after training | ☐ | ☐ | ☐ |  |
| Number of research staff supporting research capacity building activities | ☐ | ☐ | ☐ |  |
| Number of participants reporting that training materials are relevant and accessible | ☐ | ☐ | ☐ |  |
| Number of research guidelines used (internal, government, network) | ☐ | ☐ | ☐ |  |
| Number of times research findings impacted program, practice or policy | ☐ | ☐ | ☐ |  |
| Number of times research findings impacted quality of care/ health outcomes | ☐ | ☐ | ☐ |  |
| Number of research patents | ☐ | ☐ | ☐ |  |
| Number of times research findings led to reduction in costs of product / service/ intervention. | ☐ | ☐ | ☐ |  |
| Number of networks/ collaboration established or joined | ☐ | ☐ | ☐ |  |
| Number of external donors expressing interest to fund activities | ☐ | ☐ | ☐ |  |
| Number of forums between policy makers and researchers | ☐ | ☐ | ☐ |  |

Have you used other metrics for research capacity building activities?

☐ Yes ☐ No

If Yes, what was the metric and what was the output /outcome / impact?

|  | **Metric** | **output /outcome / impact** |
| --- | --- | --- |
|  |  |  |
|  |  |  |
|  |  |  |

**Section E:**

How will work move forward post-PHIT?

Do you have any other comments?
