# Supplementary material for: Research capacity building integrated into PHIT projects: leveraging research and research funding to build national capacity
Source: BMC Health Serv Res. 2017 Dec 21;17(Suppl 3):825. doi: 10.1186/s12913-017-2657-6 (PMC5763288; doi:10.1186/s12913-017-2657-6)
Supplement: Supplementary file 2 — Metrics table outlining each country’s indicator measurement. (DOCX 32 kb) [file 12913_2017_2657_MOESM2_ESM.docx]

**Additional File 2: Metrics table outlining each country’s indicator measurement**

| **Capacity building level** | **Metric** | **Ghana** | **Mozambique** | **Rwanda** | **Tanzania** | **Zambia** | **Total** | **Average** |
| --- | --- | --- | --- | --- | --- | --- | --- | --- |
|  |  | **n** | **n** | **n** | **n** | **n** | **n** | **n** |
| Individuals and teams | Average number of applications per training | 15 | NR | 30 | 11 | 15 | 71 | 18 |
|  | Average number of participants per training | 12 | NR | 15 | 11 | 8 | 46 | 12 |
|  | Number of participants completing training | NR | 54 | 28 | 66 | 53 | 201 | 50 |
|  | Number of practitioners / clinical staff trained | NR | NR | NR | 2 | 12 | 14 | 7 |
|  | Number of program staff trained | NR | NR | NR | 9 | 18 | 27 | 14 |
|  | Number of PhD / Masters / research scholarships awarded | NR | 4 | 16 | 0 | 2 | 22 | 6 |
|  | Number of mentorship relationships (individual or team-based) | NR | 11 | 9 | 36 | NR | 56 | 19 |
|  | Number of research protocols developed | NR | 8 | 29 | 2 | 5 | 44 | 11 |
|  | Number of publishable manuscripts written | NR | 12 | 62 | 25 | 17 | 116 | 29 |
|  | Number of peer reviewed publications | NR | 8 | 24 | 18 | 9 | 59 | 15 |
|  | Number of peer reviewed publications with a national first authors | NR | 1 | 10 | 11 | 7 | 29 | 7 |
|  | Number of peer reviewed publications with a national last/senior authors | NR | 0 | 16 | 0 | 2 | 18 | 5 |
|  | Number of conferences /workshop /consultancies /public lectures where trainees presented | NR | 15 | 4 | 30 | 50 | 99 | 25 |
|  | Number of trainees collaborating in new research | NR | 30 | NR | 6 | 7 | 43 | 14 |
|  | Number of trainees who led new research projects | NR | 1 | NR | NR | 3 | 4 | 2 |
|  | Number of trainees who became facilitators or mentors | NR | 4 | NR | 3 | 12 | 19 | 6 |
| Organizational | Number of research trainings conducted (whether long or short) | 11 | 27 | 6 | 6 | 4 | 54 | 11 |
|  | Average types of research trainings conducted | 5 | 12 | 4 | 6 | 4 | 25 | 6 |
|  | Range of the main trainings per session | 1 week | 3 days -2 weeks | 2-6 days | 5-10 days | 3-14  days | 2-14 days |  |
|  | Range of training/contact time in days | 60 days | 10 days | 2-6 days | 106 days | 5-150  days | 2-150 days |  |
|  | Number of PhD or Masters level trainers or facilitators | 6 | 7 | 6 | 4 | 2 | 25 | 5 |
|  | Number of national PhD or Masters trainers or facilitators | 4 | 4 | 1 | 2 | NR | 11 | 3 |
|  | Average full time research staff supporting RCB activities | 10 | 4 | NR | 6 | 5 | 25 | 6 |
|  | Average mentorship hours per mentorship relationship | NR | NR | NR | 225 | NR | 225 | 225 |
|  | Number of research related career promotions | NR | 1 | NR | 8 | NR | 9 | 5 |
|  | Number of participants reporting that training materials are relevant and accessible | NR | NR | 16 | NR | NR | 16 | 16 |
|  | Number of research guidelines used (internal, government or network) | NR | NR | 2 | NR | 6 | 8 | 4 |
| Networks | Number of networks/ collaboration established or joined | NR | 5 | NR | NR | 4 | 9 | 5 |
|  | Number of forums between policy makers and researchers | NR | 22 | 1 | 4 | 10 | 37 | 9 |
|  | Number of times research findings impacted program, practice or policy | NR | NR | NR | NR | 3 | 3 | 3 |
|  | Number of times research findings led to reduction in costs of product, service or intervention. | NR | NR | NR | NR | NR | N/A | N/A |
|  | Number of times research findings impacted quality of care or health outcomes | NR | NR | NR | NR | NR | N/A | N/A |
|  | Number of external donors expressing interest to fund activities | NR | 4 | NR | 2 | 2 | 8 | 3 |
| NR: Not reported | | | | | | | | |
